# Supplementary material for: Identification of photoperiod-induced specific miRNAs in the adrenal glands of Sunite sheep (Ovis aries)
Source: Front Vet Sci. 2022 Jul 22;9:888207. doi: 10.3389/fvets.2022.888207 (PMC9354845; doi:10.3389/fvets.2022.888207)
Supplement: Supplementary file 1 [file Data_Sheet_1.ZIP › Supplementary file/miRDeep2 file.zip!/miRDeep2 file/Know_Structure/LP42A2/oar-mir-3955.pdf]

miRBase precursor : oar-mir-3955
Total read count : 406
oar-miR-3955-5p read count : 363
oar-miR-3955-3p read count : 29
remaining reads : 14

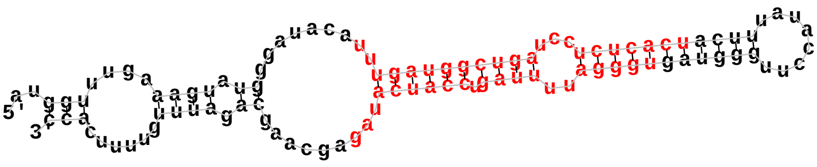

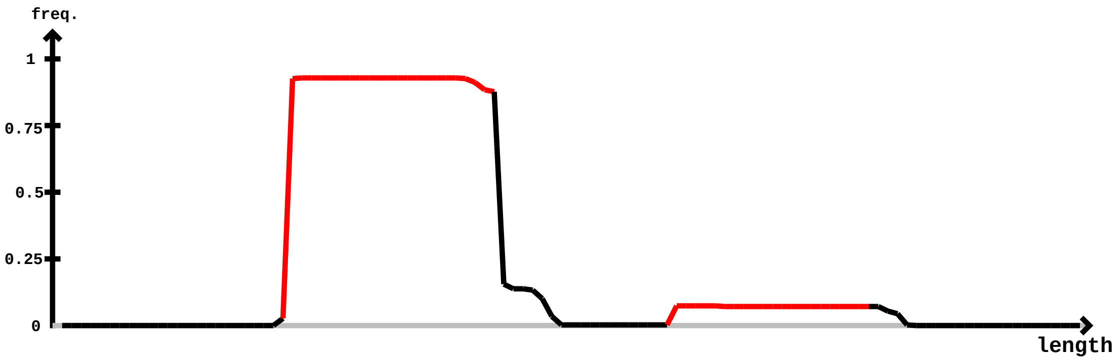

oar-miR-3955-5p

oar-miR-3955-3p

5'- augguuugaaaguaugggauacauuugauggcugauccucucacuacuuuauaccuuggguagugggauuuuaguccaucauagagcaagcagauuuguuuucacc -3'	exp		
...........((((...))))...((((((((((...(((((((((((........)))))))))))..)))).)))))).(((((((......)))))))....	reads	mm	sample
......................auuugauggcugauccucu...	1	0	seq
......................auuugauggcugauccucuca...	1	0	seq
......................auuugauggcugauccucucacu...	8	0	seq
......................auuugauggcugauccucucacuacuuua...	1	0	seq
.......................uuugauggcugauccucuc..	6	0	seq
.......................uuugauggcugauccucuca...	10	0	seq
.......................uuugauggcugauccucucac..	3	0	seq
.......................uuugauggcugauccucucacu...	283	0	seq
.......................uuugauggcugUuccucucacu...	1	1	seq
.......................uuugauggcugauccucucacua..	7	0	seq
.......................uuugauggcugauccucucacuacu..	2	0	seq
.......................uuugauggcugauccucucacuacuu...	13	0	seq
.......................uuugauggcugauccucucacuacuuu..	27	0	seq
.......................uuugauggcugauccucucacuacuuua...	12	0	seq
.......................uuugauggcugauccucucacuacuuuauaccuuggguagugggga......................................	1	0	seq
........................uugauggcugauccucucacu...	1	0	seq
....ugggauuuuaguccaucauaga.....................	7	0	seq
....ugggauuuuaguccaucauagag....................	4	0	seq
....ugggauuuuaguccaucauagagc...................	17	0	seq
....ugggauuuuaguccaucauagagca..................	1	0	seq
